# Criticality meets learning: Criticality signatures in a self-organizing recurrent neural network

**DOI:** 10.1371/journal.pone.0178683

**Published:** 2017-05-26

**Authors:** Bruno Del Papa, Viola Priesemann, Jochen Triesch

**Affiliations:** 1 Frankfurt Institute for Advanced Studies, Johann Wolfgang Goethe University, Frankfurt am Main, Germany; 2 International Max Planck Research School for Neural Circuits, Max Planck Institute for Brain Research, Frankfurt am Main, Germany; 3 Department of Non-linear Dynamics, Max Planck Institute for Dynamics and Self-Organization, Göttingen, Germany; 4 Bernstein Center for Computational Neuroscience, Göttingen, Germany; Consejo Nacional de Investigaciones Cientificas y Tecnicas, ARGENTINA

## Abstract

Many experiments have suggested that the brain operates close to a critical state, based on signatures of criticality such as power-law distributed neuronal avalanches. In neural network models, criticality is a dynamical state that maximizes information processing capacities, e.g. sensitivity to input, dynamical range and storage capacity, which makes it a favorable candidate state for brain function. Although models that self-organize towards a critical state have been proposed, the relation between criticality signatures and learning is still unclear. Here, we investigate signatures of criticality in a self-organizing recurrent neural network (SORN). Investigating criticality in the SORN is of particular interest because it has not been developed to show criticality. Instead, the SORN has been shown to exhibit spatio-temporal pattern learning through a combination of neural plasticity mechanisms and it reproduces a number of biological findings on neural variability and the statistics and fluctuations of synaptic efficacies. We show that, after a transient, the SORN spontaneously self-organizes into a dynamical state that shows criticality signatures comparable to those found in experiments. The plasticity mechanisms are necessary to attain that dynamical state, but not to maintain it. Furthermore, onset of external input transiently changes the slope of the avalanche distributions – matching recent experimental findings. Interestingly, the membrane noise level necessary for the occurrence of the criticality signatures reduces the model’s performance in simple learning tasks. Overall, our work shows that the biologically inspired plasticity and homeostasis mechanisms responsible for the SORN’s spatio-temporal learning abilities can give rise to criticality signatures in its activity when driven by random input, but these break down under the structured input of short repeating sequences.

## Introduction

A popular hypothesis states that neural circuits operate near a second order phase transition, a so called critical point [1, 2]. A critical state has also been argued to possess maximal information processing aspects including computational performance during classification tasks [3], dynamical range [4], information transmission and storage capacity [5, 6]. In order to always operate near this state, however, neural circuits should self-organize to adapt to a great variety of inputs while still maintaining all those properties. Although this precise adaptation was argued to be the result of constant plasticity action [7–10] or dynamic neuronal gains [11], the principles dominating the self-organization in the brain are still not clearly understood.

Typically, experiments in brain circuits look indirectly for a phase transition state by assessing criticality signatures. These signatures are approximate power-law distributions of the sizes and durations of neuronal avalanches, i.e. spatio-temporal clusters of spikes or other suitably defined “events”. Power-laws have been found in networks *in vitro* [1, 12–14], and in anaesthetized animals [15]. For awake mammals, criticality signatures were found for coarse measures like local field potentials (LFP), while spiking activity resembles a subcritical regime [16–19]. Last, large scale fMRI measurements also support the criticality hypothesis [20] and links between supercriticality and epileptic seizures in humans have been proposed [21].

From a theoretical perspective, many computational models have studied how neural networks can be tuned towards and away from criticality and how they are able to maintain it over time [7, 8, 22–30]. For example, deterministic networks combining short-term and long-term plasticity have been shown to have power-law distributed avalanches [9]. Typically, previous studies either described how models are tuned to criticality by plasticity rules or they investigated information processing properties of critical networks. Whether the plasticity mechanisms responsible for spatio-temporal learning also can tune a network to criticality remains an open question [31].

Here we addressed the following question: can a neural network driven by a combination of plasticity mechanisms that allow a network to learn patterns show signatures of criticality? In order to investigate network self-organization towards a regime showing stable criticality signatures, we have chosen a Self-Organizing Recurrent Neural Network, or SORN. The original SORN [32] is a network of excitatory and inhibitory binary neurons combining spike-timing dependent plasticity (STDP), with homeostatic regulation of firing thresholds and synaptic normalization. This model has been shown to possess sequence and spatio-temporal learning abilities [32, 33]. Furthermore, it has recently been used to explain a wide range of findings on spontaneous brain activity, the variability of neural responses, and the relationship between the two [34]. Beyond this, a variant of the SORN including a form of structural plasticity (SP) has been shown to reproduce the distribution and fluctuation patterns of synaptic efficacies observed in cortex and hippocampus [35] while being capable of learning an artificial grammar [36]. This model has also been demonstrated to exhibit the spontaneous formation of synfire chains [37] and a novel *deferred chaos* effect [38]. Additionally, a model from the SORN family with leaky integrate-and-fire neurons, LIF-SORN, has also been demonstrated to reproduce non-random features of cortical synaptic wiring via the interaction of the different plasticity mechanisms [39].

Here, we model the original SORN with additional plasticity mechanisms (inhibitory STDP and SP) [35] and observe power-law distributions for both duration and size of neuronal avalanches, suggesting that the SORN might self-organize into a critical state under specific membrane noise levels. Although this self-organization requires plasticity, we show that plasticity was not necessary to maintain the criticality signatures in the network’s spontaneous activity. Furthermore, we find that, while neuronal membrane noise is a requirement for the presence of the power-laws, external input can drive the network away from the putative critical regime, matching evidence found in the *ex-vivo* turtle brain [29]. The description of criticality signatures in the spontaneous activity of a recurrent network model showing learning abilities, and their break-down under external input, is unprecedented and helps to clarify how self-organization due to plasticity underlies both phenomena at the same time.

## Methods

### Recurrent network model

The model we used belongs to the self-organizing recurrent network (SORN) family of models [32] and was almost identical to the model introduced in [35], differing slightly in the synaptic normalization rule: in addition to the normalization of incoming excitatory connections, we added a separate normalization of incoming inhibitory connections, in agreement with experimental evidence [40]. It is important to point out that both the model in [35] and our SORN model had three additional features when compared to the original SORN model [32]: the action of inhibitory spike-timing dependent plasticity (iSTDP), a structural plasticity mechanisms (SP) and the addition of neuronal membrane noise. Those features are described in detail in the following paragraphs.

Our SORN was composed of a set of threshold neurons divided into *N*^*E*^ excitatory and *N*^*I*^ inhibitory units, with *N*^*I*^ = 0.2 × *N*^*E*^. The neurons were connected through weighted synapses *W*_*ij*_ (going from unit *j* to unit *i*), which were subject to synaptic plasticity. The network allowed connections between excitatory neurons *W*^*EE*^, from excitatory to inhibitory neurons *W*^*IE*^, and from inhibitory to excitatory neurons *W*^*EI*^, while connections between inhibitory neurons and self-connections were absent. Each neuron *i* had its own threshold, which did not vary with time for the inhibitory neurons, $T_{i}^{I}$, and was subject to homeostatic plasticity for the excitatory neurons, $T_{i}^{E}\left(t\right)$.

The state of the network, at each discrete time step *t*, was given by the binary vectors **x**(*t*) ∈ {0, 1}^*N*^*E*^^ and **y**(*t*) ∈ {0, 1}^*N*^*I*^^, corresponding to the activity of excitatory and inhibitory neurons, respectively. A neuron would fire (“1” state) if the input received during the previous time step, a combination of recurrent synaptic drive, membrane noise $\xi_{i}^{E/I}$ and external input $u_{i}^{\text{Ext}}$, surpassed its threshold. Otherwise it stayed silent (“0” state), as described by:

$$\begin{array}{c} x_{i}\left(t+1\right)=\Theta \left[\sum_{j=1}^{N^{E}}W_{ij}^{EE}\left(t\right)x_{j}\left(t\right)-\sum_{k=1}^{N^{I}}W_{ik}^{EI}\left(t\right)y_{k}\left(t\right)+u_{i}^{Ext}\left(t\right)+\xi_{i}^{E}\left(t\right)-T_{i}^{E}\left(t\right)\right], \end{array}$$ (1)

$$\begin{array}{c} y_{i}\left(t+1\right)=\Theta \left[\sum_{j=1}^{N^{E}}W_{ij}^{IE}\left(t\right)x_{j}\left(t\right)+\xi_{i}^{I}\left(t\right)-T_{i}^{I}\right], \end{array}$$ (2)

in which Θ is the Heaviside step function. Unless stated otherwise, *ξ* represents the unit’s independent Gaussian noise, with mean zero and variance *σ*^2^ = 0.05, and was interpreted as neuronal membrane noise due to the extra input from other brain regions not included in this model. The external input *u*^*Ext*^ was zero for all neurons, except during the external input experiment and the learning tasks, in which subsets of units received supra-threshold input at specific time steps. Each time step in the model represented the time scale of STDP action, being roughly in the 10 to 20 ms range.

The synaptic weights and neuronal thresholds were initialized identically to previous works [35, 38]: *W*^*EE*^ and *W*^*EI*^ started as sparse matrices with connection probability of 0.1 and 0.2, respectively, and *W*^*IE*^ was a fixed fully connected matrix. The three matrices were initialized with synaptic weights drawn from a uniform distribution over the interval [0, 0.1] and normalized separately for incoming excitatory and inhibitory inputs to each neuron. The thresholds *T*^*I*^ and *T*^*E*^ were drawn from uniform distributions over the intervals $\left[0,T_{\text{max}}^{I}\right]$ and $\left[0,T_{\text{max}}^{E}\right]$, respectively, with $T_{\text{max}}^{I}=1$ and $T_{\text{max}}^{E}=0.5$. After initialization, the connections and thresholds evolved according to five plasticity rules, detailed below. It is important to highlight that the connectivity between excitatory neurons varied over time due to the action of plasticity on *W*^*EE*^.

First, excitatory to excitatory connections followed a discrete *spike-timing dependent plasticity rule* (STDP) [41]:

$$\begin{array}{c} \Delta W_{ij}^{EE}\left(t\right)=\eta_{\text{STDP}}\left[x_{i}\left(t\right)x_{j}\left(t-1\right)-x_{j}\left(t\right)x_{i}\left(t-1\right)\right]. \end{array}$$ (3)

The rule increased the weight $W_{ij}^{EE}$ by a fixed small quantity *η*_STDP_ every time neuron *i* fired one time step after neuron *j*. If neuron *i* fired one step before neuron *j*, the weight was decreased by the same amount. Negative and null weights were pruned after every time step.

Second, inhibitory to excitatory connections were subject to a similar rule, the inhibitory STDP (iSTDP). It played a role in balancing the increase of activity due to STDP and regulating the overall network activity. Every time an inhibitory neuron *j* fired one time step before an excitatory neuron *i*, the connection $W_{ij}^{EI}$, if existent, was increased by *η*_inh_/*μ*_IP_, in which *μ*_IP_ represented the desired average target firing rate for the network (given as a parameter to the model). However, if the synapse was successful (*i.e.*, if neuron *j* firing kept neuron *i* silent in the next time step), $W_{ij}^{EI}$ was reduced by a bigger value *η*_inh_. These rules could be simply written as:

$$\begin{array}{c} \Delta W_{ij}^{EI}\left(t\right)=-\eta_{\text{inh}}y_{j}\left(t-1\right)\left[1-x_{i}\left(t\right)\left(1+1/\mu_{\text{IP}}\right)\right]. \end{array}$$ (4)

Third, both *W*^*EE*^ and *W*^*EI*^ were subject to yet another form of plasticity, the synaptic normalization (SN). It adjusted the incoming connections of every neuron in order to limit the total input a neuron could receive from the rest of the network, thus limiting the maximum incoming recurrent synaptic signal. This rule did not regulate the relative strengths of the connections (shaped by both STDP and iSTDP), but the total amount of input each neuron receives. SN could be written as an update equation, applicable to *W*^*EE*^ and *W*^*EI*^, and executed at each time step after all other synaptic plasticity rules:

$$\begin{array}{c} W_{ij}\left(t\right)\leftarrow W_{ij}\left(t\right)/\sum_{j}W_{ij}\left(t\right). \end{array}$$ (5)

Fourth, the structural plasticity (SP) added new synapses between unconnected neurons. It added a random directed connection between two unconnected neurons (at a particular time step) with a small probability *p*_SP_, simulating the creation of new synapses in the cortex. The probability was set to *p*_SP_(*N*^*E*^ = 200) = 0.1 for a network of size *N*^*E*^ = 200, and *p*_SP_ scaled with the square of the network size:

$$\begin{array}{c} p_{\text{SP}}\left(N^{E}\right)=\frac{N^{E}\left(N^{E}-1\right)}{200\times 199}p_{\text{SP}}\left(200\right) \end{array}$$ (6)

The new synapses were set to a small value *η*_SP_ = 0.001, and while most of them were quickly eliminated due to STDP action in the subsequent time steps, the life-times of active synapse followed a power-law distribution [35].

Last, an *intrinsic plasticity* (IP) rule was applied to the excitatory neurons’ thresholds. To maintain an average firing rate for each neuron, the thresholds adapted at each time step relying on a homeostatic plasticity rule, keeping a fixed target firing rate *H*_IP_ for each excitatory neuron. The target firing rate, unless stated otherwise, was drawn from a normal distribution $H_{\text{IP}}\approx N\left(\mu_{\text{IP}},\sigma_{\text{IP}}^{2}\right)$. However, for simplicity, it could be set to the network average firing rate *μ*_IP_, thus being equal for all neurons [35]. We set *μ*_IP_ = 0.1 and $\sigma_{\text{IP}}^{2}=0$, or equivalently, 10% of active excitatory neurons (on average) fire per time step. Assuming one time step equals 10 to 20 ms, these constants resulted in an average firing rate in the 5 − 10 Hz range.

$$\begin{array}{c} \Delta T_{i}^{E}=\eta_{\text{IP}}\left[x_{i}\left(t\right)-\mu_{\text{IP}}\right]. \end{array}$$ (7)

Unless stated otherwise, all simulations were performed with the learning rates from [35]: *η*_STDP_ = 0.004, *η*_inh_ = 0.001 and *η*_IP_ = 0.01.

### Phases of network development

**Fig 1:**
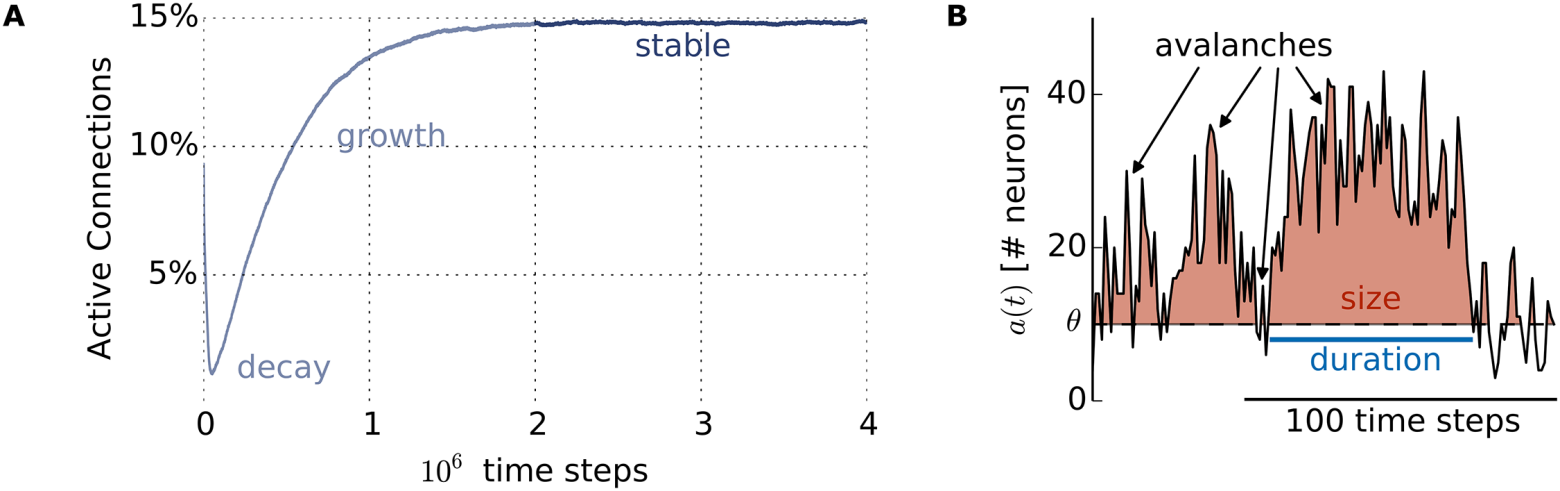
SORN regimes and neuronal avalanches. (A) Fraction of active connections in the SORN, starting from a random connected graph with 0.1 active connections. It exhibits three self-organization phases: *decay*, *growth*, and *stable*. (B) Activity threshold *θ* for a typical snapshot of SORN activity *a*(*t*) (150 time steps). Avalanche duration is indicated in blue and avalanche size in shaded red. The neuronal avalanches were measured only during the *stable* phase.

As observed before [35], the spontaneous activity of the SORN showed three different self-organization phases regarding the number of active excitatory to excitatory synapses when driven only by Gaussian noise (Fig 1A). After being randomly initialized, the number of active connections fell quickly during the first 10^5^ time steps (the *decay* phase) before slowly increasing (*growth* phase) until stabilizing after around two million time steps (*stable* phase), where only minor fluctuations are present. In order to avoid possible transient effects, we concentrated our analyses only on the *stable* phase, discarding the first 2 × 10^6^ time steps. In this sense, we measured neuronal avalanches in the regime into which the SORN self-organizes driven only by membrane noise and its own plasticity mechanisms.

### Neuronal avalanches definition via activity threshold

It is important to highlight that the SORN is fundamentally different from classical self-organizing critical models such as the Bak-Tang-Wiesenfeld Sandpile model [42] or branching processes regarding the lack of separation of time scales, *i.e.* no pause is implemented between any two avalanches [43] (see also the discussion in [18]). Importantly, such a separation of time scales also does not apply to neural activity *in vivo*. Each SORN neuron could receive input from other neurons, the noisy drive *ξ*, and an additional input (during the extra input experiment), all of which occurred at every time step.

Motivated by those fundamental differences, a distinct definition of neuronal avalanches based on thresholding the neural activity has been used in a previous model [25]. Similarly, we introduced here a threshold *θ* for the network activity *a*(*t*):

$$\begin{array}{c} a\left(t\right)=\sum_{i=0}^{N_{E}}x_{i}\left(t\right) \end{array}$$ (8)

In more detail, a constant background activity *θ* was subtracted from *a*(*t*) for all time steps *t*, allowing for frequent silent periods and neuronal avalanches’ measurements. *θ* was set to half of the mean network activity 〈*a*(*t*)〉, which by definition is 〈*a*(*t*)〉 = *μ*_IP_ = 0.1. For simplicity, *θ* was rounded to the nearest integer, as *a*(*t*) can only assume integer values. Each neuronal avalanche could be described by two parameters: its duration *T* and size *S*. An avalanche started when the network activity went above *θ*, and *T* was the number of subsequent time steps during which the activity remained above *θ*. *S* was the sum of spikes exceeding the threshold at each time step during the avalanche (Fig 1B, red area). More specifically, for an avalanche starting at the time step *t*_0_, *S* was given by:

$$\begin{array}{c} S=\sum_{t=t_{0}}^{t_{0}+T}\left(a\left(t\right)-\theta \right) \end{array}$$ (9)

As the activity included all the network’s neurons, subsampling effects [19, 44, 45] could be ruled out. Furthermore, as the target firing rate was *H*_IP_ = 0.1, 10% of the excitatory neurons were active, on average, at every time step, which made quiescent periods a rare occurrence.

### External input

In order to study the effects of external input on the SORN self-organization, we chose an adapted version of a condition previously designed to investigate neural variability and spontaneous activity in the SORN [32, 34]. The condition consisted of presenting randomly chosen “letters” repeatedly to the network (*i.e.*, at each time step, a random “letter” was chosen with equal probability and presented to the network). In our case, we chose a total of 10 different letters. Each letter gave extra input to a randomly chosen, non-exclusive subset of *U*^*E*^ = 0.02 × *N*^*E*^ excitatory neurons, closely following a previous probabilistic network model [29]. The subsets corresponding to each letter were fixed at the beginning and kept identical until the end of each simulation. Neurons which did not receive any input had $u_{i}^{\text{Ext}}\left(t\right)=0$ for all *t*, while neurons matched with a specific letter received a large additional external input $u_{i}^{\text{Ext}}\left(t\right)=10^{7}$ at the time step in which the letter was presented, making sure that the neuron spiked.

We followed the approach introduced in a previous experimental procedure in the turtle visual cortex [29]: the SORN was initially simulated up until the stable phase (2 × 10^6^ time steps), when external input was turned on and neuronal avalanches were measured during a transient period and after readaptation. A single neuronal avalanche was considered part of the transient period if it started during the first 10 time steps after external input onset. According to our time step definition due to STDP action, this transient window was roughly in the 100 − 200 ms range, approximately the same time window employed for the experimental data [29]. After the transient period, neuronal avalanches were again measured for 2 × 10^6^ time steps after readaptation.

### Learning tasks

We analyzed the network performance and the occurrence of the aforementioned criticality signatures in two simple learning tasks, which allowed us to compare our results to previous work [32].

The first task was a Counting Task (CT), introduced in [32], in which a simpler SORN model (without the iSTDP and SP mechanisms and membrane noise) has been shown to outperform static reservoirs. The CT consisted of a random alternation of structured inputs: sequences of the form “ABBB…BC” and “DEEE…EF”. Each sequence was shown with equal probability and contained *n* + 2 “letters”, with *n* repetitions of the middle letters “B” or “E”. Each letter shown to the network represented the activation of a randomly chosen, non-exclusive subset of *U*^*E*^ excitatory neurons at the time step in which it was shown.

The second task, which we call Random Sequence Task (RST), consisted also in the reproduction of “letters” of a large sequence of size *L*, initially chosen at random from an “alphabet” of *A*_*S*_ different letters. The same random sequence was repeated during a single simulation, but different simulations received different random sequences as input. This task definition allowed not only for the description of the SORN’s learning abilities under a longer, more variable input but also, in the case of large *L*, for the analysis of criticality signatures under an approximately random input.

For both tasks, the SORN performance was evaluated as in [32]. Starting from the random weight initialization, we simulated the network for *T*_plastic_ = 5 × 10^4^ time steps with all plasticity mechanisms active. The performance was evaluated by training a layer of readout neurons for *T*_train_ = 5000 time steps in a supervised fashion (using the Moore-Penrose pseudo-inverse method) and measured the correct prediction of the next input letter. The input at time step *t* was predicted based on the network internal state *x*′(*t*), calculated similarly to Eq (1), but ignoring the *u*(*t*) input term. The performance was calculated based on a sample of additional *T*_test_ = 5000 time steps for both tasks. For CT, however, we ignored the first letter of each sequence during the performance calculation, as the two sequences are randomly alternated.

The additional free parameters included in the simulation of the learning tasks were chosen based on previous SORN implementations: *U*^*E*^ = 0.05 × *N*^*E*^ and *A*_*S*_ = 10. The membrane noise was kept as Gaussian noise, with standard deviation *σ* = 0.05. Additionally, for the CT, we also looked at the performance in the case of no membrane noise (*σ* = 0) and of no iSTDP and SP action, in order to have a direct comparison between this model and the original SORN model [32].

### Power-law fitting and exponents

The characterization of power-law distributions may be affected by large fluctuations, especially in their tails, which leads to common problems ranging from inaccurate estimates of exponents to false power-laws [46]. In our model, in order to fit the neuronal avalanche distributions of duration *T* and size *S* and calculating their exponents *α* and *τ*, respectively:

$$\begin{array}{c} f\left(T\right)∼T^{-\alpha }, \end{array}$$ (10)

$$\begin{array}{c} f\left(S\right)∼S^{-\tau }, \end{array}$$ (11)

we relied on the *powerlaw* python package [47]. The package fits different probability distributions using maximum likelihood estimators. We used exponential binning when plotting the avalanche distributions, with exponential bin size *b*_*s*_ = 0.1 (the measurement of the exponents did not depend on the particular bin choice). Additionally, even though the left cut-offs of our data were *f*(*T*) = 1 and *f*(*S*) = 1, those points were not visible in the plots due to the binning, which considered the bin centers. We compared different distributions provided by the package, of which pure power-laws provided the best fit, but for simplicity only pure power-laws and power-laws with exponential cutoffs are shown in the results (see S1 Table for a comparison of parameters). In order to account for finite size effects in the pure power-law fits, the exponents for duration *α* and size *τ* were estimated between a minimum *X*_min_ and a maximum *X*_max_ cutoff, with *X* ∈ {*T*, *S*}. For the majority of our results (SORN with *N*^*E*^ = 200 and *N*^*I*^ = 40), we used the following parameters: *T*_min_ = 6, *T*_max_ = 60, *S*_min_ = 10, *S*_max_ = 1500, chosen based on the goodness of the power-law fit. The maximum cutoff was scaled accordingly for bigger networks. For the power-law with exponential cutoff, we kept the same *X*_min_ and removed *X*_max_:

$$\begin{array}{c} f\left(x\right)∼x^{-\alpha^{*}}e^{-\beta^{*}x}. \end{array}$$ (12)

with α* being the power-law exponent and β* the exponential cut-off.

The ratio between the power-law distributions’ exponents, $\frac{\alpha -1}{\tau -1}$ is also predicted by renormalization theory to be the exponent of the average size of avalanches with a given duration 〈*S*〉(*T*):

$$\begin{array}{c} \left\langle S\right\rangle \left(T\right)∼T^{\frac{\alpha -1}{\tau -1}}. \end{array}$$ (13)

This positive power-law relation is obeyed by dynamical systems exhibiting crackling noise [48] and has been also found in *in vitro* experiments [13].

## Results

As criticality has been widely argued to be present in biological neural systems, we first identified the presence of its most common signature in a recurrent network shaped by biologically inspired plasticity mechanisms. We showed that neuronal avalanches with power-law distributed durations and sizes appear after network self-organization through plasticity.

We then described how synaptic plasticity and units’ membrane noise are necessary for the emergence of the criticality signatures. In agreement with experimental evidence [29], we also verified that while random external input can break down the power-laws, subsequent adaptation is able to bring the network back to a regime in which they appear. Last, we showed that the same power-laws break down under simple structured input of sequence learning tasks.

### SORN shows power-law avalanche distributions

**Fig 2:**
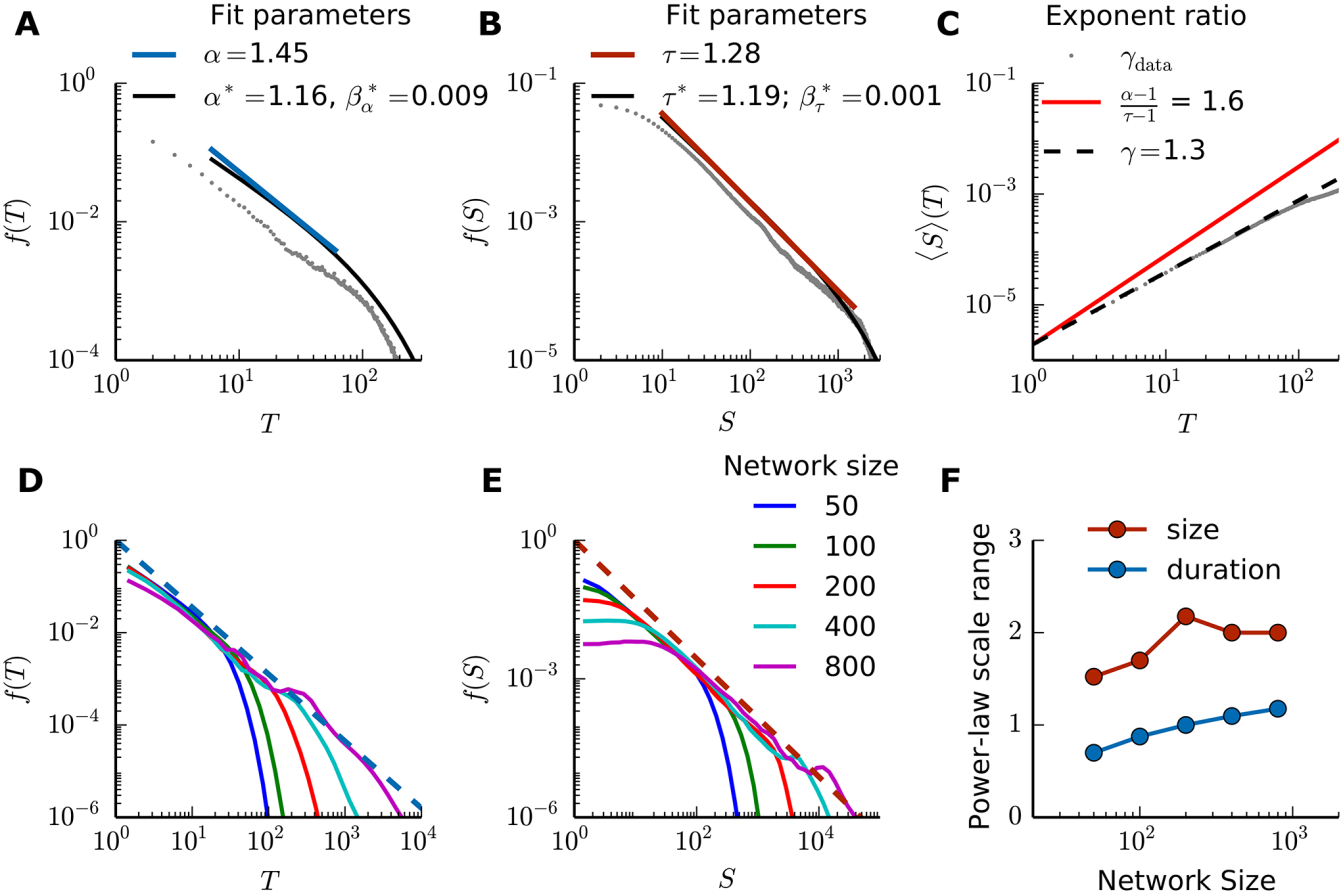
Power-law distributed neuronal avalanches in the SORN’s stable phase. (A), (B) Normalized distributions of duration *T* and size *S* of neuronal avalanches, respectively, for *N*^*E*^ = 200. The raw data points of 50 independent SORN simulations are shown in gray. The power-law fit is shown in blue/red and the power-law with exponential cut-off fit is shown in black for comparison. (C) Avalanches’ average size 〈*S*〉(*T*) as a function of duration, for simulated data (gray) and theoretical prediction (red). The dashed black line shows a power-law with exponent *γ* = 1.3, approximately fitting the raw data from SORN simulations. (D), (E) Scaling of avalanches’ distributions for networks of different sizes. Dashed lines show the exponents *α* and *τ* calculated from pure power-laws for *N*^*E*^ = 200 (shown in the top row). (F) Power-law range for networks of different sizes, obtained by estimating the cut-off point. All distributions show combined data of 50 independent simulations.

We simulated a network of *N*^*E*^ = 200 excitatory and *N*^*I*^ = 40 inhibitory neurons for 5 × 10^6^ time steps. The neuronal avalanches were measured after the network self-organization into the stable phase, and the activity threshold *θ* was fixed as half of the mean activity of the SORN. Both neuronal avalanche duration *T* (Fig 2A) and size *S* (Fig 2B) distributions were well fitted by power-laws, but for different ranges. For the size, the power-law distribution fitted approximately two orders of magnitude, while the duration is only well fitted for approximately one before the cut-off. The faster decay observed in the distribution’s tails could not be fitted by a power-law with exponential cut-offs, and was hypothesized to be the result of finite size effect. Indeed, with increasing network size the power-law distributions extended over larger ranges (Fig 2D–2F), and the exponents remained roughly the same (avalanche’s duration: *α* ≈ 1.45; avalanche’s size: *τ* ≈ 1.28). Thus, both for simplicity and for a reduced simulation time, we kept the SORN size constant for the rest of the results (*N*^*E*^ = 200, *N*^*I*^ = 40).

The expected relation between the scale exponents $\frac{\alpha -1}{\tau -1}$ from Eq (13) inferred from the power-law fitting, however, did not match the exponents obtained from the avalanche raw data (Fig 2C), although the average avalanche size did follow a power-law as a function of avalanche duration, with exponent *γ*_data_ ≈ 1.3. It is worth noting that, although the predictions were not compatible, our numerical exponent *γ*_data_ agreed with the one calculated directly from experimental data from cortical activity in a previous experimental study [13].

**Fig 3:**
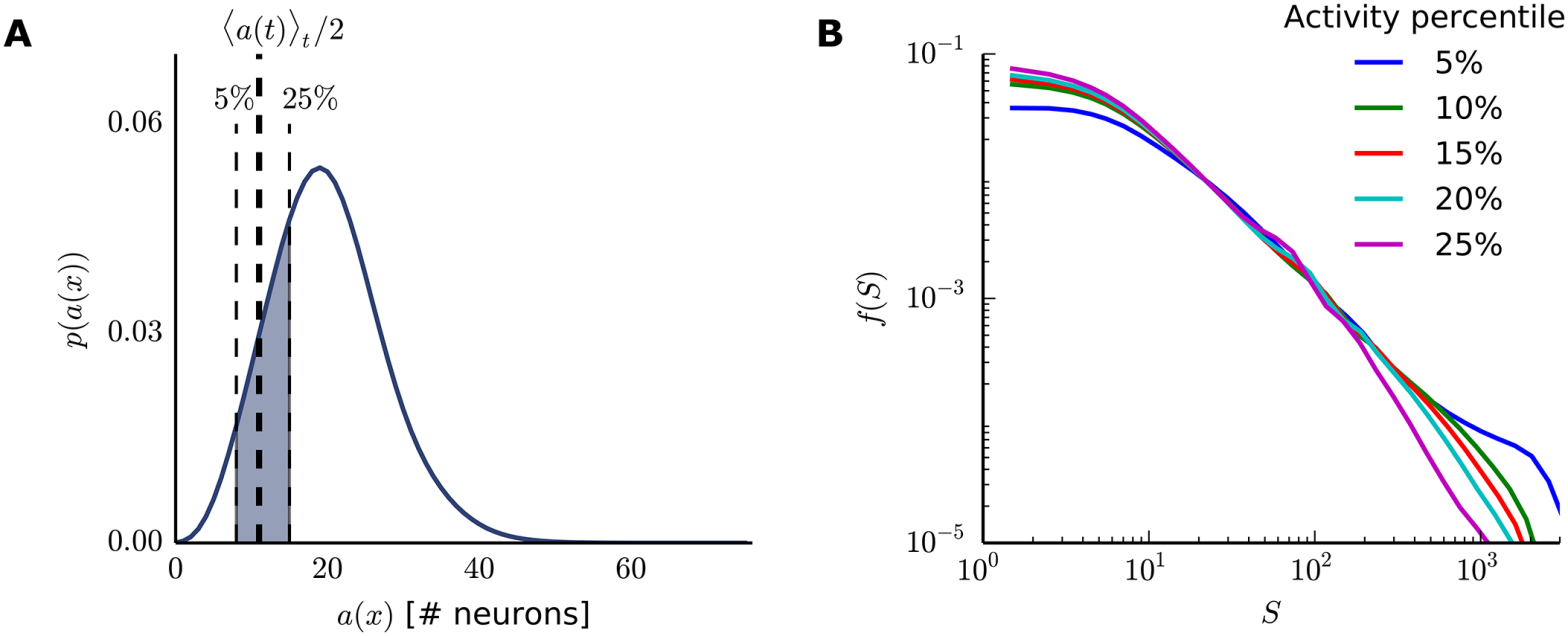
Robustness to choice of activity threshold. (A) Activity distribution function for a SORN with *N*^*E*^ = 200. The shaded area shows the approximate region where the power-laws hold. The activity peak, as expected due to the target firing rate, is 10% of the number of excitatory neurons. (B) Avalanche size distribution for different activity thresholds *θ* set as activity percentiles. Although showing different exponents, the power-laws hold for different thresholds (as seen, for example, for *θ* set at the 5th or 10th percentiles of the activity distribution). Curves show combined data from 50 simulations.

The activity threshold *θ*, which defines the start and end of avalanches, should in principle affect the avalanches’ distributions since the slope of the power-laws might depend on its precise choice. Small thresholds should increase the avalanches’ duration and size while reducing the total number of avalanches. Large thresholds are expected to reduce the avalanche durations and sizes while also reducing the number of avalanches. An adequate threshold *θ* has been suggested as half of the network mean activity 〈*a*(*t*)〉_*t*_ [25], which we have been using so far in this work. While different thresholds resulted in different exponents (see S2 Table for the range of estimated exponents for *T* and *S*), power-law scaling was robust for a range of *θ* values, roughly between 10% and 25% activity percentiles (Fig 3). This window contained the previously used half mean activity 〈*a*(*t*)〉_*t*_/2 (roughly 10% activity percentile for a network of size *N*_*E*_ = 200). Therefore, we could verify that the avalanche definition in terms of *θ* was indeed robust enough to allow for a clear definition of power-law exponents. The unusual left cut-off for the avalanche size, observed independently of the threshold value, was arguably a consequence of our avalanche size definition, Eq (9). In particular, removing the explicit dependence on *θ* changed the left cut-off shape, but did not affect the power-laws ranges or exponents (see S1 Fig for one example of alternative avalanche size definition).

### Criticality signatures are not the result of ongoing plasticity

**Fig 4:**
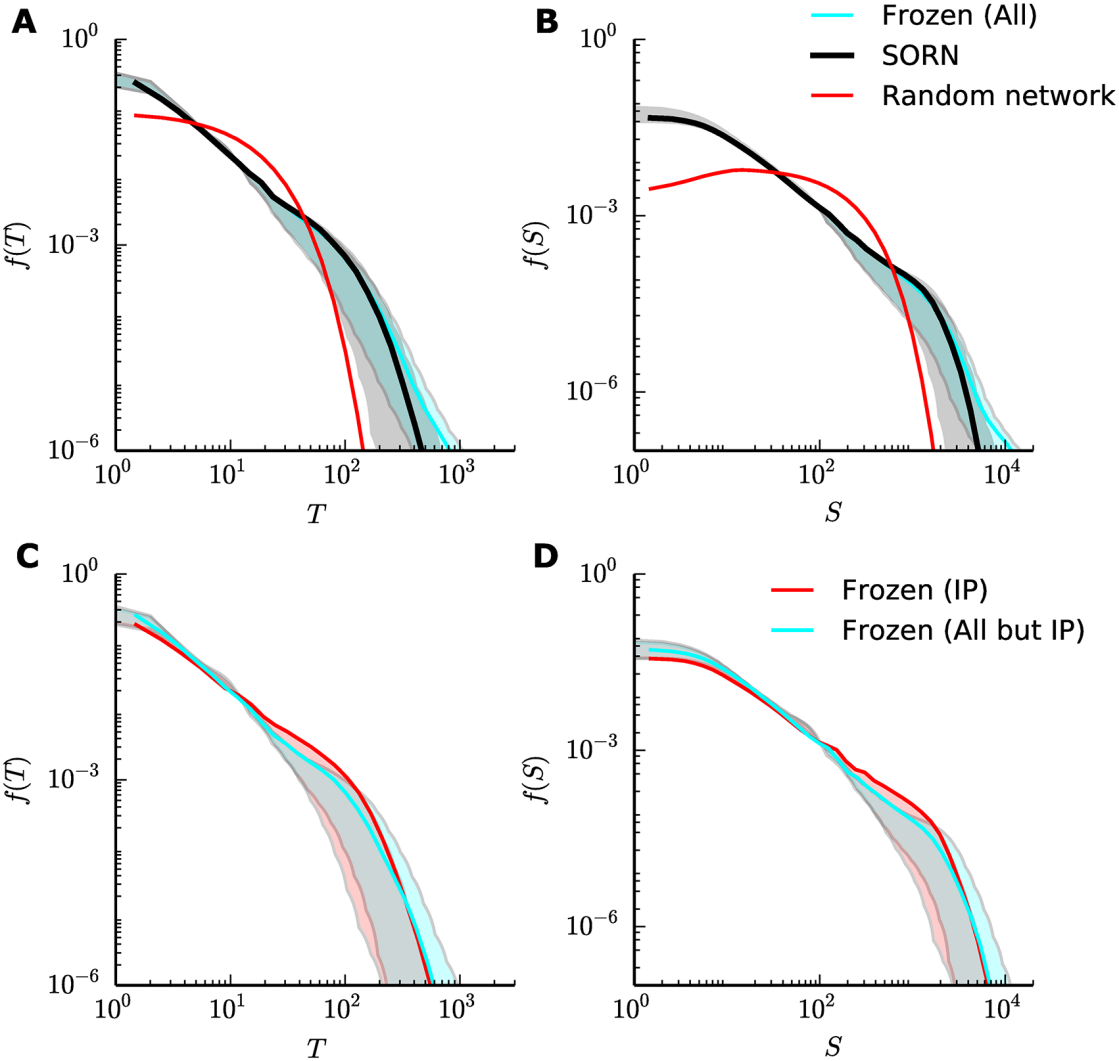
SORN with frozen plasticity. (A), (B) Distribution of avalanche durations and sizes, respectively, for *N*^*E*^ = 200 units, comparing typical SORNs (black), randomly initialized SORNs without plasticity action (red) and SORNs with all five plasticity mechanisms frozen at the stable phase (cyan). (C), (D) Distribution of avalanche durations and sizes, respectively, for the same network size, comparing SORNs with frozen IP (red) and frozen STDP, iSTDP, SN and SP (cyan) at the stable phase. Curves are combined data from 50 independent simulations, and shaded regions show the effects of variations in the activity threshold (*θ* between the 5th and 25th percentiles of the activity distribution).

We investigated the role of the network plasticity on the signatures of criticality. The first question we asked is whether plasticity is necessary to drive the SORN into a regime where it shows signatures of criticality, or if they also appear right after random initialization. Thus, we compared our results to a SORN with no plasticity action, which is equivalent to a randomly initialized network. The avalanche distributions observed in the random networks, for both duration and size, did not show power-laws, as shown in Fig 4A and 4B (red curves), resembling exponential distributions rather than power-laws and indicating that plasticity was indeed necessary for the self-organization.

After verifying that the combination of plasticity mechanisms was indeed necessary to drive the network from a randomly initialized state towards a state in which the power-laws appear, we asked whether this result is purely due to the continued action of such mechanisms. If the power-laws appear only when plasticity is active, they could be a direct result of the ongoing plasticity. If the power-laws hold even when all plasticity is turned off after self-organization, this supports the interpretation that the plasticity mechanisms drive the network structure to a state where the network naturally exhibits criticality signatures. We compared, therefore, our previous results with the distributions found for a *frozen SORN*: a network where all plasticity mechanisms where turned off after self-organization.

The SORN was simulated up until the stable phase, when the simulation was divided in two: a normal SORN and the *frozen SORN*. We used the same random seed for the membrane noise in both cases (Gaussian noise with mean zero and variance *σ*^2^ = 0.05), so that differences due to noise are avoided. Furthermore, initialization bias could also be ruled out as the networks had the same initialization parameters and thus were identical up to the time step when plasticity was turned off. The *frozen SORN* resulted in virtually identical power-law distributions for durations and sizes (Fig 4, top row), and the only significant differences were observed in their tails. With frozen plasticity, an increase in the number of large avalanches was observed. This effect can be partly explained by the absence of homeostatic mechanisms that control network activity in the normal SORN. Likewise, freezing individual mechanisms (as for example, the IP) did not affect the overall avalanche duration and size distributions (Fig 4, bottom row, and S2 Fig), indicating that they were not the result of continued action of any particular plasticity rule from the model.

Taken together, these results showed that the SORN’s plasticity mechanisms allowed the network to self-organize into a regime where it showed signatures of criticality. However, the continued action of the plasticity mechanisms was not required for maintaining these criticality signatures, once the network has self-organized.

### Noise level contributes to the maintenance of the power-laws

The standard deviation *σ* of the membrane noise *ξ* was one of the model parameters that influenced the SORN’s dynamics. Therefore, our next step was to investigate whether the criticality signatures depend on the distribution of *ξ* and its standard deviation *σ*.

**Fig 5:**
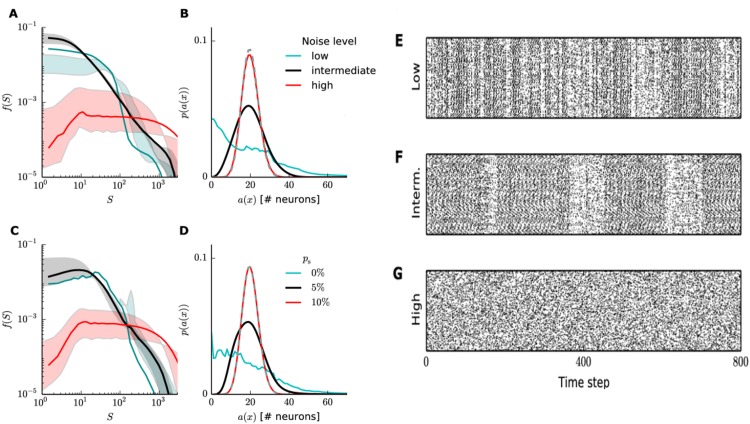
Noise level influences the SORN dynamical regime. Left, top row: avalanches’ size (A) and activity (B) distributions for SORN with different Gaussian noise levels: low (*σ*^2^ = 0.005), intermediate (*σ*^2^ = 0.05) and high (*σ*^2^ = 5). Very weak or strong noise levels break down the power-laws, suggesting a different non-critical regime. Left, bottom row: Avalanches’ size (C) and activity (D) distributions for the *random spike* noise source (see text), showing a similar effect. Gray dashed lines are binomial distributions (*n* = *N*_*E*_ = 200, *p* = *μ*_IP_ = 0.1), the theoretical prediction for independent neurons, and shaded areas show the effects of variations in the activity threshold (*θ* between the 5th and 25th percentiles of the activity distribution). All curves show combined data of 50 independent simulations. (E), (F), (G) Typical raster plots of excitatory unit activity at low, intermediate and high Gaussian noise levels, respectively, for *N*_*E*_ = 200.

As expected, we found that the avalanche and activity distributions suggested three different regimes, represented here by three different levels of noise. In the case of high noise levels (*σ*^2^ = 5), the neurons behaved as if they were statistically independent, thus breaking down the power-laws and showing binomial activity centered at the number of neurons expected to fire at each time step (*i.e.* the mean of the firing rate distribution *H*_IP_). Low noise levels resulted in a distribution of avalanche sizes resembling a combination of two exponentials, while the activity occasionally died out completely for periods of a few time steps. A close look at the raster plots of excitatory neuronal activity (Fig 5E–5G) also revealed that large bursts of activity only happened at intermediate noise levels, while low noise levels resulted mostly in short bursts and high noise levels resulted in Poisson-like activity. Therefore, we concluded that, together with the plasticity mechanisms, the noise level determined the network dynamical regime. The activity distribution (Fig 5B) supported the hypothesis of a phase transition, as it went from a binomial distribution for high noise levels to a distribution with faster decay and maximum near zero for lower noise levels.

To further investigate the contribution of noise to the maintenance of the criticality signatures, we tested if other types of noise could have a similar effect on the network’s dynamic regime, and how diffused this noise needed to be in order to allow for the appearance of the power-laws. First, we switched from Gaussian noise to *random spikes*: each neuron received input surpassing its threshold with a small probability of spiking *p*_s_ at each time step. Using *p*_s_ as a control parameter in the same way as the Gaussian noise variance, we could reproduce all the previous findings: three different distribution types and a transition window, in which the power-law distributions of neuronal avalanches appear (Fig 5C).

Last, we found that limiting the noise action to a subset of units, while keeping all plasticity mechanisms on, abolishes power laws completely (see S3 Fig). Different subset sizes were compared (10%, 5% and 0% of the excitatory units were continuously active), and the activity threshold *θ* was set again to 〈*a*(*t*)〉_*t*_/2, but now excluding the subset of continuously active units. We concluded that the power-laws require not only a specific noise level, but also noise distribution across the network units.

### Network readaptation after external input onset

We tested whether the onset of external input is able to break down the power-laws we have measured so far. Experimental evidence suggests a change in power-law slope in the transient period after onset of an external stimulus [29]. This work proposed that network readaptation due to short term plasticity brings the criticality signatures back after a transient period, implying self-organization towards a regime in which power-laws appear.

Our version of external input consisted of random “letters”, each of which activated a subset of *U*^*E*^ excitatory neurons. We compared neuronal avalanche distributions in two different time periods: directly after external input onset and after network readaptation by plasticity. The activity threshold *θ* was kept the same for both time periods.

**Fig 6:**
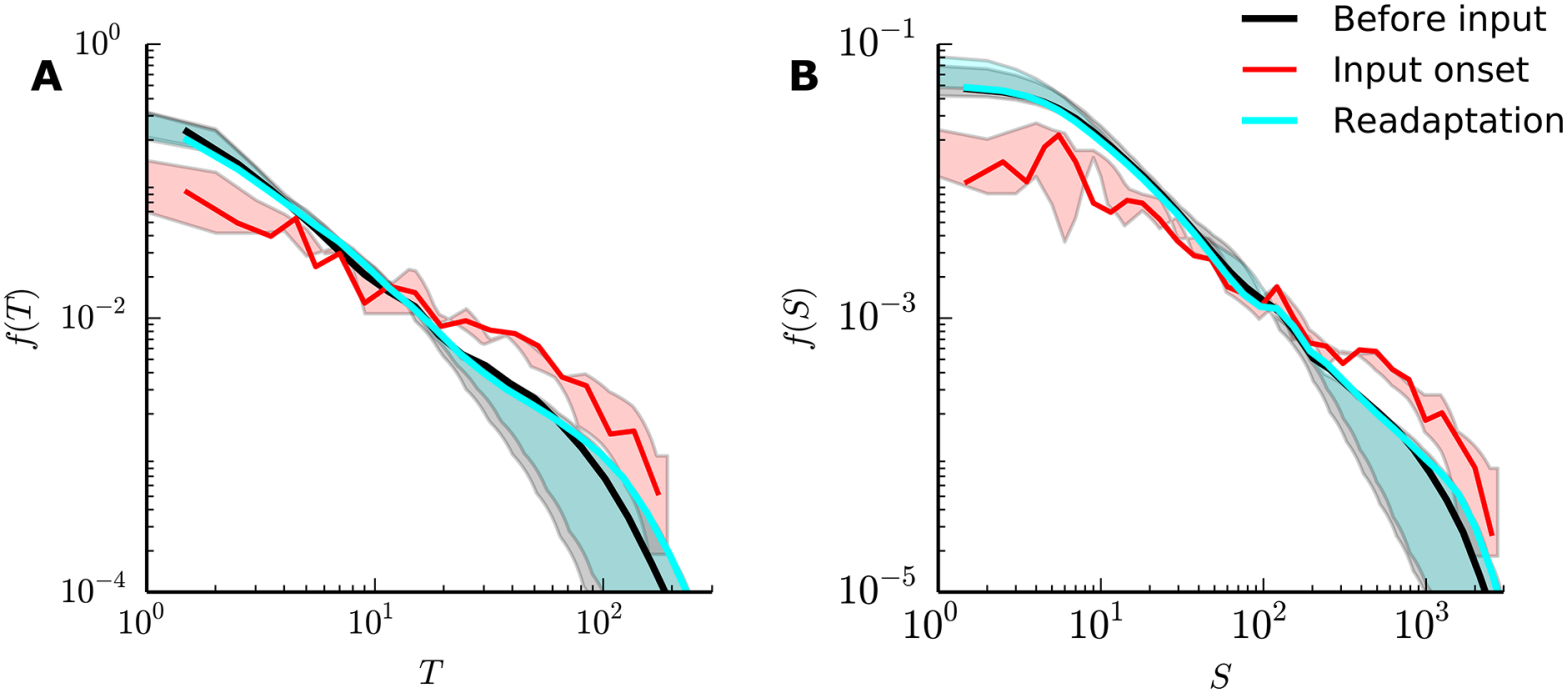
SORN readaptation under external input. (A), (B) Duration and size distributions, respectively, after external input onset: transient readaptation period (red) and remaining 2 × 10^6^ time steps (cyan). Before input and Readaptation curves show combined data from 50 independent simulations. Input onset curves show data from 250 input onset trials, and shaded regions show the effects of variations in the activity threshold (*θ* between the 5th and 25th percentiles of the activity distribution).

The results agreed with the experimental evidence (Fig 6): an external input resulted in flatter power-laws (Fig 6, red curve), in agreement with experimental observations (Fig 1 in [29]). As in the experiment, we also observed a readaptation towards the power-laws, after a transient period (Fig 6, cyan curve). Furthermore, the flatter power-laws and the subsequent readaptation also appeared under weaker external inputs ($u_{i}^{\text{Ext}}∼1$). This finding supported the hypothesis that plasticity was responsible for driving the SORN towards a critical regime, even after transient changes due to external stimulation.

### Absence of criticality signatures under structured input in simple learning tasks

So far, we have observed criticality signatures in the model’s spontaneous activity and activity when submitted to a random input. We focus now on the activity under structured input of two learning tasks: a Counting Task (CT) [32] and a Random Sequence Task (RST). For details on their implementation, see the *Learning tasks* subsection.

**Fig 7:**
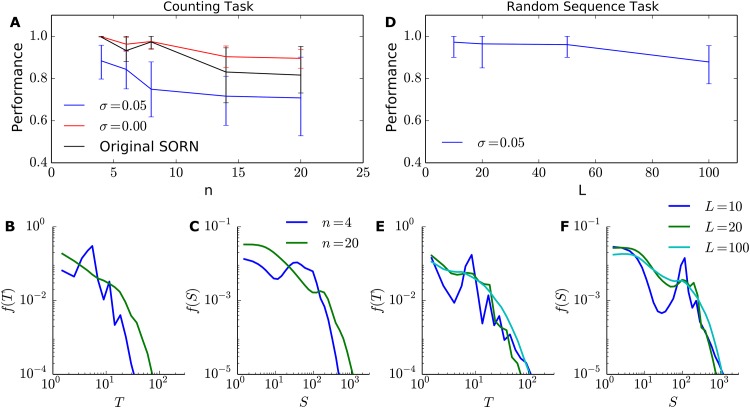
Learning tasks. (A) SORN performance for the Counting Task for sequences of different sizes, with (blue) and without (red) membrane noise. Original SORN refers to the model without iSTDP, SP and membrane noise, as introduced in [32](B), (C) Duration and size distributions, respectively, during the Counting Task for different input sequence sizes *n* (in the presence of membrane noise). (D) SORN performance for the Random Sequence Task for sequences of different sizes. (E), (F) Duration and size distributions, respectively, during the Random Sequence Task for different input sequence lengths *L*. Curves show the average of 50 independent simulations and error bars show the 5%–95% percentile interval.

In the CT, the external structured input consisted of randomly alternated sequences of “ABBB…BC” and “DEEE…EF”, with *n* middle repeated letters. Differently from the former random external input, these sequences were presented during the whole simulation, one letter per time step. First, we measured the avalanche distributions for duration and size and verified that the power-laws did not appear in this case, independently of *n* (Fig 7A and 7B), although the distributions appeared smoother and more similar to power-laws for large values of *n*. This finding suggested that structured input did not allow for the appearance of the power-laws, and in this case our plasticity mechanisms could not drive the network towards the supposed critical regime. Second, we measured the performance of the SORN in the CT by training a readout layer and calculated its performance in predicting the input letter of the next time step. We found that our model was capable of maintaining a performance higher than 90% when the membrane noise was removed (*σ* = 0), which is consistent with the results obtained in the original SORN model for the same task [32]. With the addition of membrane noise (*σ* = 0.05), however, we saw a decay in the overall performance, particularly for long sequences.

In the RST, a different form of external input was used: in the beginning of each simulation, we defined a random sequence of size *L*, which would be repeated indefinitely. We observed that under this type of input the power-laws again did not appear (Fig 7E and 7F), but, as observed in the CT, longer sequences showed smoother curves. The performance, however, stayed above ∼88% for *L* ≤ 100, demonstrating that our SORN implementation is capable of learning random sequences.

In summary, both learning tasks highlighted our model’s learning abilities and showed that the addition of plasticity mechanisms (iSTDP and SP) to the original SORN [32] does not breakdown its learning abilities. The presence of membrane noise, however, diminished the overall model performance for the CT. Furthermore, we showed that the structured input of both learning tasks was sufficient to break down the power-law distributions of avalanche size and duration.

## Discussion

The hypothesis of criticality in the brain as discussed here, which states that neural circuits possess dynamics near a phase transition state, is largely based on experimental measurements of power-law distributed neuronal avalanches. This hypothesis, however, is still very controversial, in particular because power-law distributions can be generated by a number of other mechanisms but criticality [43], for example by thresholding activity of certain kinds of stochastic systems or superposition of exponentials [49, 50]. Thus power-law scaling of physical quantities is not sufficient to demonstrate criticality. For that reason, our avalanche analysis alone is not sufficient to prove that the SORN self-organizes towards a critical point. Instead, we highlight that the combination of plasticity mechanisms in the model is sufficient to produce the same criticality signatures typically observed in experiments, independently of the question whether these systems are critical or not.

Our results suggest that the combination of biologically inspired Hebbian and homeostatic plasticity mechanisms is responsible for driving the network towards a state in which power-law distributed neuronal avalanches appear, but such plasticity action is not required for the maintenance of this state. The power-law distributions of avalanche durations and sizes in the SORN’s spontaneous activity replicate a widely observed phenomenon from cultured cortical networks [1, 13, 51] to awake animals [18, 52, 53]. Notably, the network also reproduces the short transient period with bigger and longer neuronal avalanches and subsequent readaptation after external input onset, which has been observed in the visual cortex of a turtle brain [29]. Our results are also in line with previous observations of power-laws in the externally driven case [54].

Additional previous studies have already identified plasticity mechanisms that tune a network to criticality. For example, networks of spiking neurons with STDP [8, 24] and a model of anti-Hebbian plasticity [55] showed critical dynamics. The earliest example of self-organization towards criticality in plastic neural networks is probably the network by Levina et al., who made use of dynamical synapses in a network of integrate-and-fire neurons [7, 23]. Furthermore, it is known that networks without plasticity can be fine-tuned to a critical state, where they show favorable information processing properties, both in deterministic [5, 22, 56] and stochastic [12, 25, 57] systems, or they can attain states close to criticality, e.g. operate on a Widom line [27] or a Griffith phase [58]. Those models are very important to describe the properties of a network already in a critical state. Beyond those results, here we have shown for the first time criticality signatures arising in a network model designed for sequence learning via a combination of Hebbian and homeostatic plasticity mechanisms.

The SORN’s criticality signatures, in the form of avalanche distributions, were best fit by power-laws (see S1 Table). The measured exponents for duration and size, *α* = 1.45 and *τ* = 1.28, were both smaller than those expected for random-neighbor networks (2 and 3/2, respectively). This discrepancy may be due to the fact that the SORN has a complex dynamic topology that differs from a random network after self-organization. The power-laws typically spanned one or two orders of magnitude for the durations and sizes, respectively, which is comparable to experimental data. Before and after the power-law interval, the size distribution often showed a right *and* a left cutoff. While the right cutoff typically arises from finite size effects [59], the left cutoff is not characteristic for classical critical systems such as the branching network [60], possibly being the result of our avalanche definition based on thresholding the network activity. However, left cutoffs have been observed for neural avalanche distributions in cortex (e.g. [18, 29]). Therefore, the SORN avalanche distributions are indeed compatible with experimental ones.

The SORN was initially conceived combining biologically inspired plasticity mechanisms (STDP, IP and SN) and has been shown to outperform static reservoirs in sequence learning tasks such as the Counting Task (CT) [32]. We showed that the addition of two other plasticity mechanisms (iSTDP and SP) [35] not only was able to reproduce the previous results but also increased the performance on the CT for large sequences. The addition of membrane noise, however, lowered the overall performance, particularly for bigger sequences in this particular task. Interestingly, previous work has shown that a SORN model with such addition is capable of solving a challenging grammar learning task [36]. In our experiments, even though a specific level of membrane noise led to the appearance of criticality signatures (*σ* ≈ 0.05), the same noise level did not increase the model’s learning abilities for simple tasks when compared to the noise-free case.

While our model showed criticality signatures in its spontaneous activity, the activity under structured external input when performing learning tasks did not lead to power-law distributions of avalanche size and duration, arguably driving the network away from a critical regime. Despite the computational advantages of critical dynamics in models, subcritical dynamics may be favorable *in vivo* (see discussion in [18]), because *in vivo* subcriticality allows for a safety margin from the unstable, supercritical regime, which has been associated with epileptic seizures [21]. Interestingly, it seems that learning of patterns and structured input may bring a network to such a regime that does not show power-law distributed neuronal avalanches, which has also been observed for cortical activity of behaving animals [18].

Note that here the term ‘criticality signatures’ refers to power-law scaling for avalanche durations and sizes, a notion of criticality inspired by Bak, Tang & Wiesenfeld [42] and widely observed in experiments [1]. This ‘avalanche criticality’ may differ from other critical phase transitions, e.g. the transition between order and chaos [18]. It is remarkable that, nonetheless, our results are consistent with those of perturbation analyses of the SORN that also suggested that with learning of structured input the network deviates from a critical state [32].

The extent to which the criticality signatures may be important for the development of learning abilities in recurrent networks is a topic for future studies. It has been argued that criticality is beneficial for information processing [3, 56], which suggests that this state may also have advantages for learning. However, our finding that the level of membrane noise necessary for the occurrence of power-laws leads to suboptimal performance in simple learning tasks suggests that the relationship between criticality and learning may be more complex.

## Supporting information

S1 FigAlternative size definition and binning.(A) Example of avalanche size distribution and power-law fit for an alternative avalanche size definition: $S^{\prime }=\sum_{t_{0}}^{t_{0}+T}a\left(t\right)$. The main effect of removing the explicit dependence of *S* on *θ* is seen before the left cut-off. The power-law exponent *τ*, however, remains largely unaffected, *τ* ≈ 1.3 (compare to Fig 2B). (B) Effects of exponential binning in the avalanche distributions. Changing the exponential bin size *b*_*s*_ does not result in changes of the exponents. Results are shown for a network of *N*^*E*^ = 200, combining data from 50 independent simulations.(TIFF)

S2 Fig Additional distributions of duration and size for the SORN with partially frozen plasticity. (A), (B) Distributions of avalanche durations and sizes, respectively, for a network of size *N*^*E*^ = 200, comparing a typical SORN (black) with a SORN with frozen iSTDP (blue) and frozen STDP, SN, IP and SP (cyan). (C), (D) Distributions of avalanche durations and sizes, respectively, for a network of the same size, now comparing a typical SORN (black) with a SORN with frozen STDP and SP (blue) and frozen iSTDP and IP (cyan). Results are combined data from 36 independent simulations.
(TIFF)

S3 Fig Limiting noise to a subset of excitatory neurons breaks down the power-laws. (A), (B) Distributions of avalanches’ size and activity, respectively, for SORN with noise limited to randomly chosen subsets of excitatory neurons. Percentages indicate the percent of excitatory units receiving the *random spike* noise at each time step. All curves show combined data of 100 independent simulations, with *θ* set at 〈*a*(*t*)〉_*t*_/2 (after removal of the always active neurons).
(TIFF)

S1 Table Fit parameters for Fig 2 (*N*^*E*^ = 200, *N*^*I*^ = 40). Comparison between exponential and power-law fits for the curves in Fig 2A and 2B (raw data from 50 independent SORN trials with 10^6^ time steps each). The goodness of fit *R* is the loglikelihood ratio between power-laws and the indicated distributions (a positive *R* means that data is more likely power-law distributed, while a negative *R* means the compared distribution is more likely a better fit). For further details, check the *powerlaw* package detailed description [47].
(TIFF)

S2 Table Exponents *α* and *τ* for different activity thresholds *θ* (*N*^*E*^ = 200, *N*^*I*^ = 40). Power-law exponents for duration and size for the activity thresholds *θ* described in Fig 3. *R*_exp_ is the goodness of fit (loglikelihood ratio between a power-law and an exponential fit) in each case [47].
(TIFF)
