# Supplementary figures and images for: Criticality meets learning: Criticality signatures in a self-organizing recurrent neural network
Source: PLoS One. 2017 May 26;12(5):e0178683. doi: 10.1371/journal.pone.0178683 (PMC5446191; doi:10.1371/journal.pone.0178683)

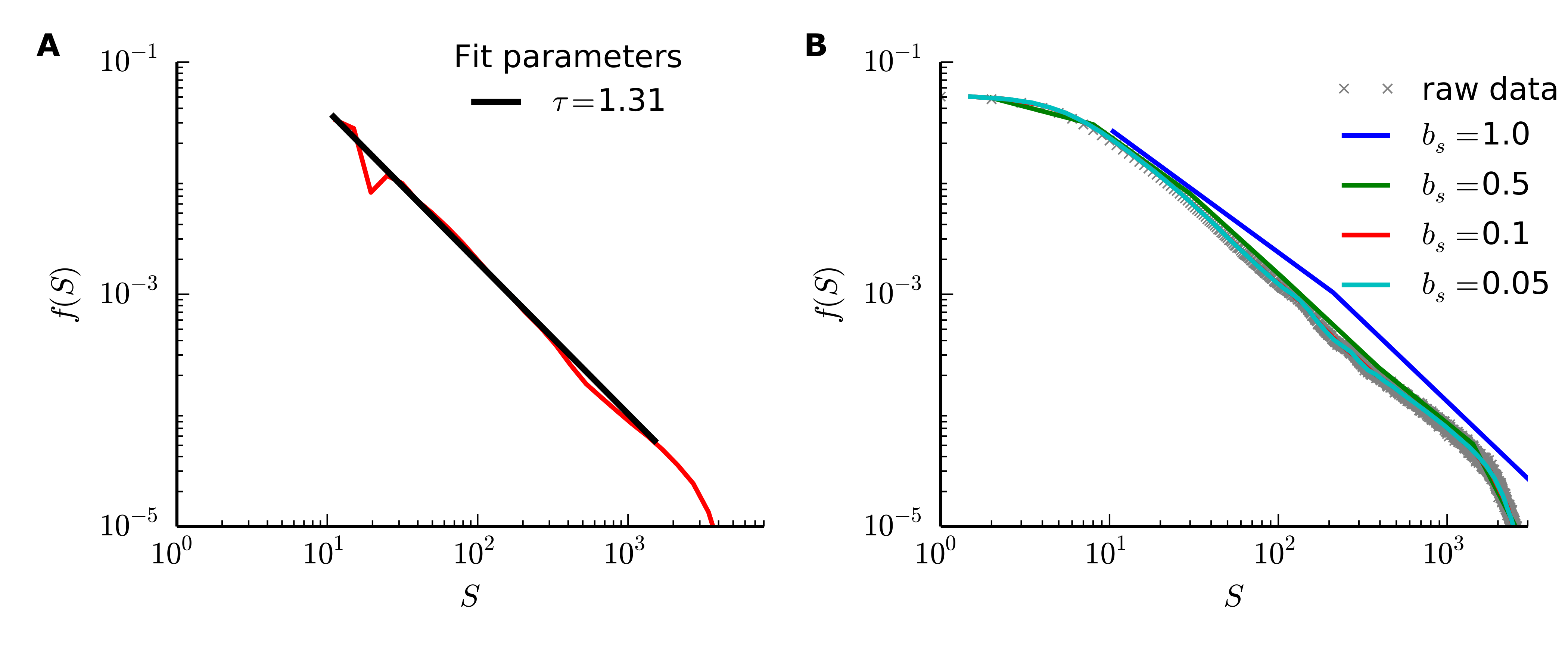

Supplement: S1 Fig — (A) Example of avalanche size distribution and power-law fit for an alternative avalanche size definition: S′=∑t0t0+Ta(t). The main effect of removing the explicit dependence of S on θ is seen before the left cut-off. The power-law exponent τ, however, remains largely unaffected, τ ≈ 1.3 (compare to Fig 2B). (B) Effects of exponential binning in the avalanche distributions. Changing the exponential bin size bs does not result in changes of the exponents. Results are shown for a network of NE = 200, combining data from 50 independent simulations. (TIFF) [file pone.0178683.s001.tiff]

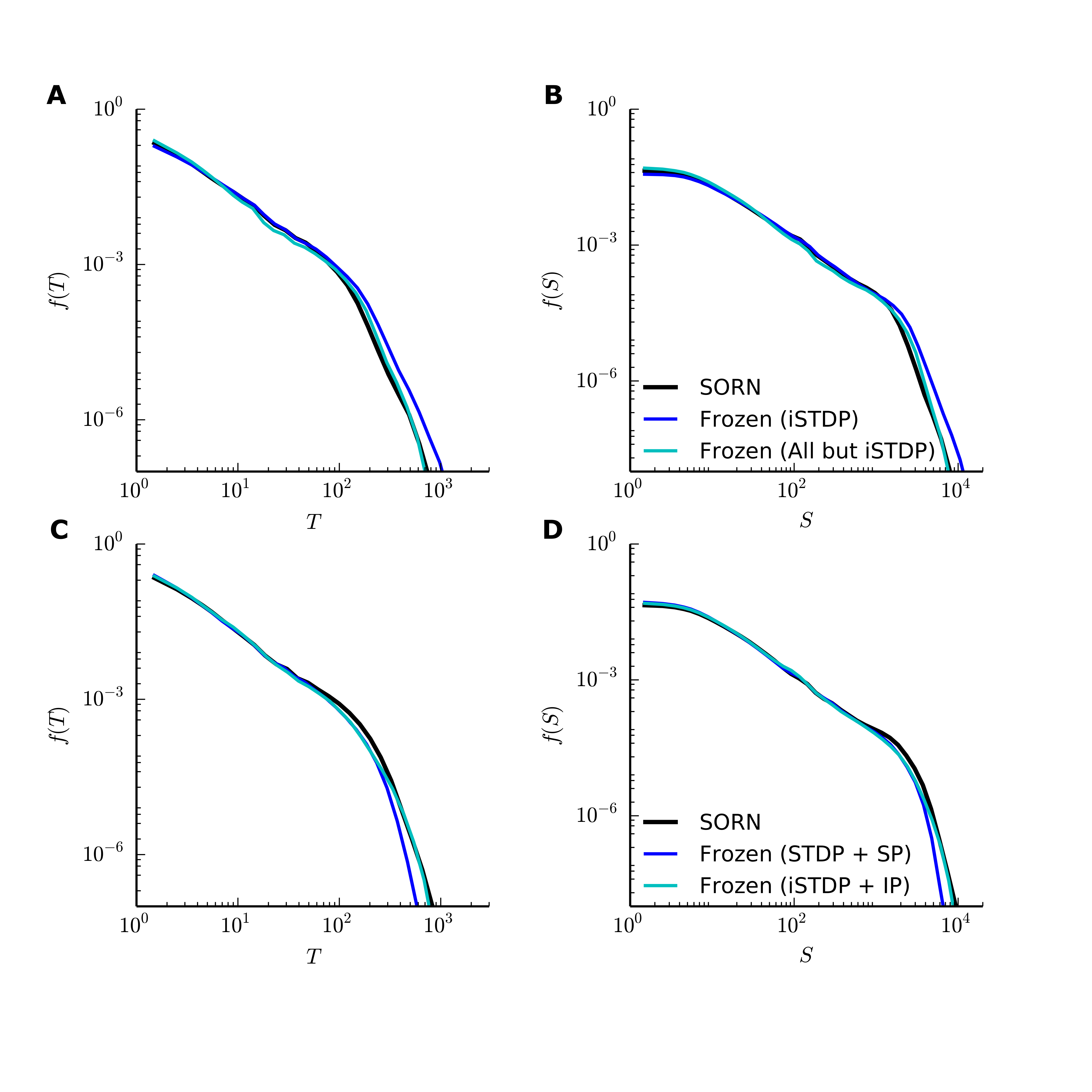

Supplement: S2 Fig — (A), (B) Distributions of avalanche durations and sizes, respectively, for a network of size NE = 200, comparing a typical SORN (black) with a SORN with frozen iSTDP (blue) and frozen STDP, SN, IP and SP (cyan). (C), (D) Distributions of avalanche durations and sizes, respectively, for a network of the same size, now comparing a typical SORN (black) with a SORN with frozen STDP and SP (blue) and frozen iSTDP and IP (cyan). Results are combined data from 36 independent simulations. (TIFF) [file pone.0178683.s002.tiff]

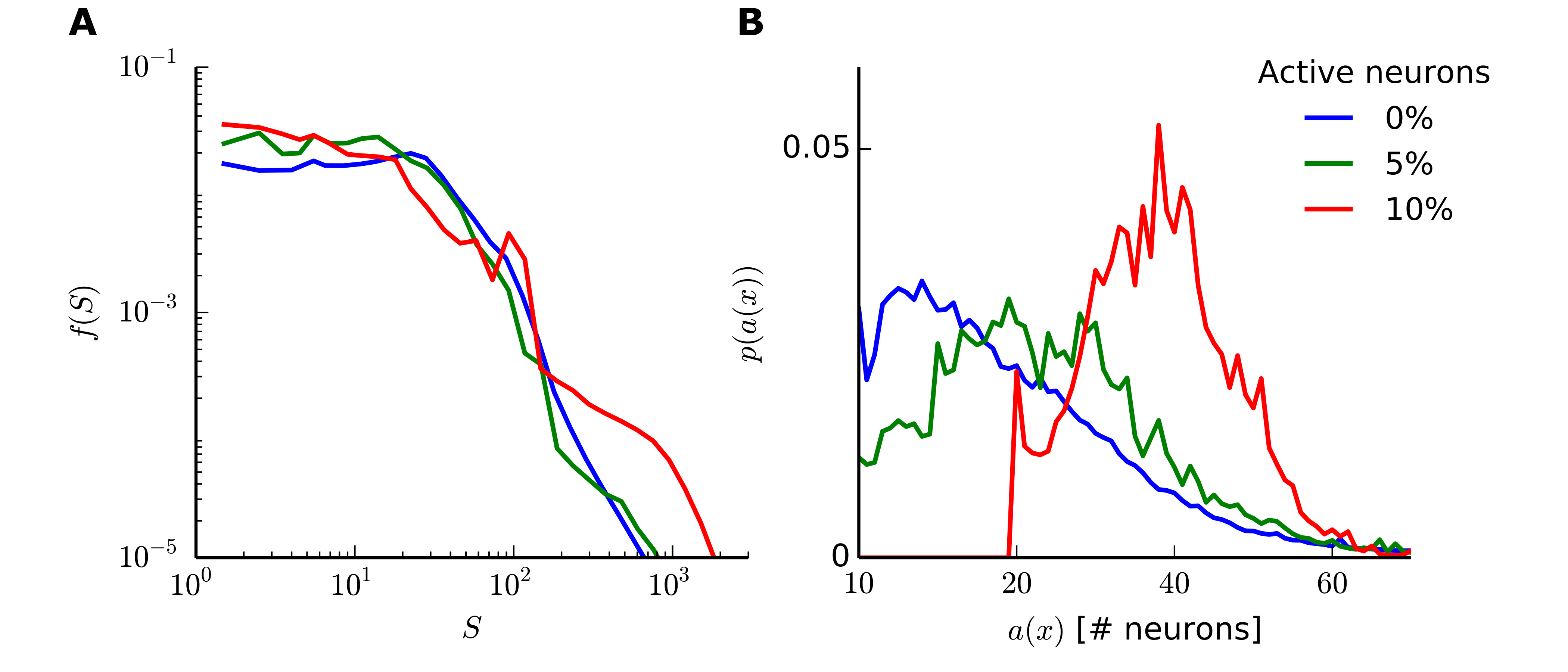

Supplement: S3 Fig — (A), (B) Distributions of avalanches’ size and activity, respectively, for SORN with noise limited to randomly chosen subsets of excitatory neurons. Percentages indicate the percent of excitatory units receiving the random spike noise at each time step. All curves show combined data of 100 independent simulations, with θ set at 〈a(t)〉t/2 (after removal of the always active neurons). (TIFF) [file pone.0178683.s003.tiff]

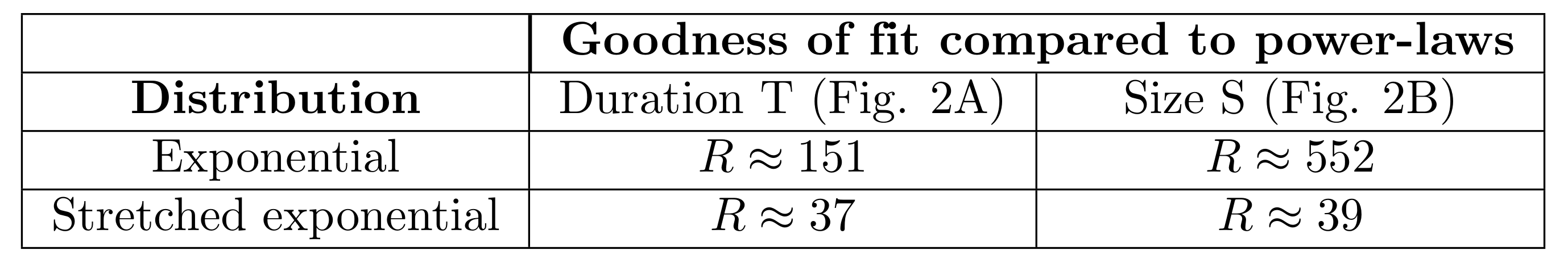

Supplement: S1 Table — Comparison between exponential and power-law fits for the curves in Fig 2A and 2B (raw data from 50 independent SORN trials with 106 time steps each). The goodness of fit R is the loglikelihood ratio between power-laws and the indicated distributions (a positive R means that data is more likely power-law distributed, while a negative R means the compared distribution is more likely a better fit). For further details, check the powerlaw package detailed description [47]. (TIFF) [file pone.0178683.s004.tiff]

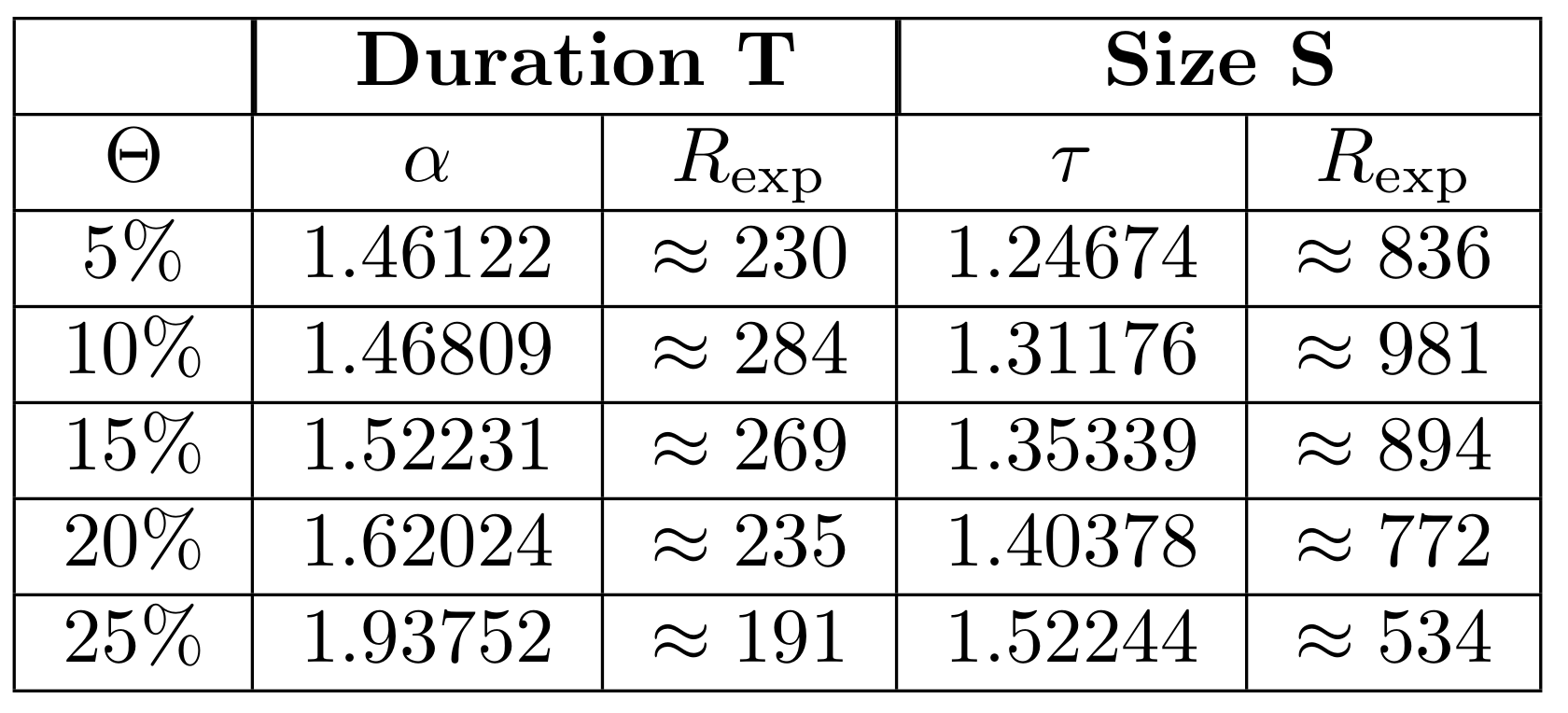

Supplement: S2 Table — Power-law exponents for duration and size for the activity thresholds θ described in Fig 3. Rexp is the goodness of fit (loglikelihood ratio between a power-law and an exponential fit) in each case [47]. (TIFF) [file pone.0178683.s005.tiff]
